# Cold induced pain elicits reproducible breath metabolomic responses across geographically distinct populations

**DOI:** 10.1016/j.isci.2026.115857

**Published:** 2026-04-22

**Authors:** Mélina Richard, Kapil Dev Singh, Dilan Sezer, Sarah Buergler, Luana Palermo, Yannick Schulz, Zhifeng Tang, Xin Luo, Urs Frey, Philippe C. Cattin, Xue Li, Jens Gaab, Pablo Sinues

**Affiliations:** 1 University Children’s Hospital Basel (UKBB), Spitalstrasse 33, 4031 Basel, Switzerland; 2 Department of Biomedical Engineering, University of Basel, Hegenheimermattweg 167b, 4123 Allschwil, Switzerland; 3 Faculty of Psychology, University of Basel, Missionsstrasse 60/62, 4055 Basel, Switzerland; 4 College of Environment and Climate, Institute of Mass Spectrometry and Atmospheric Environment, Guangdong Provincial Key Laboratory of Speed Capability Research, Jinan University, Guangzhou 510632, China; 5 University Hospital of Child and Adolescent Psychiatry and Psychotherapy, University of Bern, Bern, Switzerland

**Keywords:** human physiology, clinical neuroscience, systems biology, metabolomics, omics, machine learning

## Abstract

Observer-independent assessment of nociceptive states is particularly important in non-communicative or vulnerable patient populations, where self-report is unavailable. In this proof-of-concept study, we explored whether exhaled breath metabolomics, measured in real time using secondary electrospray ionization high-resolution mass spectrometry (SESI-HRMS), can capture rapid physiological responses to the cold pressor test (CPT), a standardized model of acute sympathetic and nociceptive activation. Forty healthy adults (*n* = 19 in Switzerland, *n* = 21 in China) underwent four breath measurements: two pre- and two post-CPT intervention. Mass spectral data revealed a reproducible and rapid metabolic shift, with over 400 upregulated features consistently observed across both cohorts. Pathway enrichment and correlation analyses implicated amino acid metabolism (e.g., glutamate, arginine, and threonine) and stress-related pathways (e.g., creatine, butanoate, and citric acid cycle intermediates). A neural network classifier distinguished pre-vs. post-CPT states (AUC = 0.856, 78% accuracy). Our findings support real-time breath analysis as a non-invasive method for detecting short-term physiological responses.

## Highlights

• Cold pressor test induces rapid, reproducible changes in the exhaled breath metabolome
• Pain-induced breath metabolite upregulation is replicated across two independent cohorts
• Altered signatures converge on amino acid metabolism, energy, and stress pathways
• Breath fingerprints enable machine learning classification of pre- and post-pain states

## Graphical abstract

**Figure:**
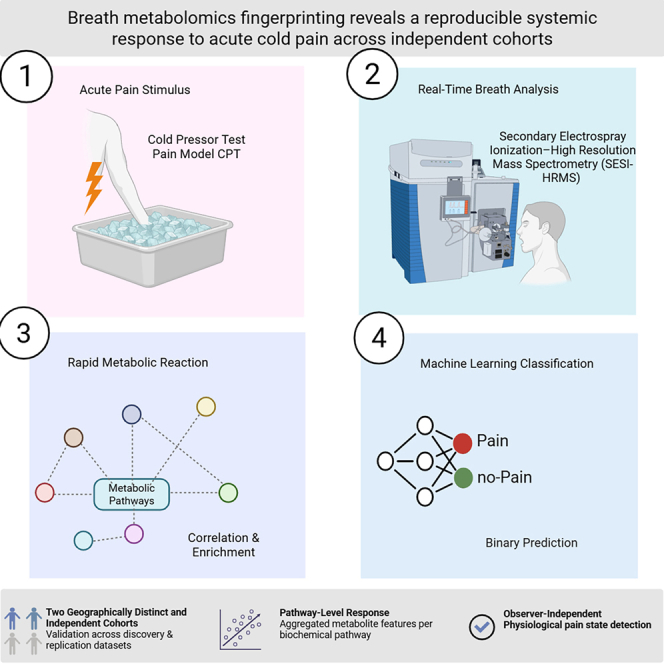

## Introduction

Effective management of nociceptive conditions remains a clinical challenge with major implications for healthcare systems and patient well-being.^1^^,^^2^ Such conditions account for high healthcare costs and frequent primary care visits and emergency consultations.^3^^,^^4^^,^^5^ Despite international guidelines, pain assessment still relies on subjective, observer-dependent tools.^6^^,^^7^ The International Association for the Study of Pain defines pain as “an unpleasant sensory and emotional experience associated with actual or potential tissue damage”,^8^ but this remains difficult to assess in non-communicative populations. Children,^9^ elderly individuals,^10^ patients with cognitive impairment,^11^ or unconscious patients^12^ are particularly vulnerable to undertreatment, not due to lack of nociceptive responses but due to limited capacity to express them. Observer-independent tools targeting underlying biological mechanisms may improve assessment and care.

In pediatric and cognitively impaired populations, inadequate pain management remains widespread,^9^^,^^11^ and in elderly patients, objective indicators are often lacking, leading to systematic underestimation.^10^ In ICUs, up to 70% of patients may experience untreated pain, with significant physiological and psychological consequences.^12^ Physiological pain responses often overlap with stress and autonomic arousal, complicating the identification of pain-specific biomarkers. In surgery and recovery settings, rapid pain detection tools are also needed.^13^^,^^14^ Skin conductance (SC) has been proposed as an observer-independent indicator, but results have been inconsistent.^15^^,^^16^^,^^17^

To address these challenges, reproducible experimental paradigms such as the cold pressor test (CPT) can help dissect the biological underpinnings of pain. CPT induces acute pain by immersing the hand or forearm in ice water (∼2°C).^18^ While initially designed to study cardiovascular reactivity,^19^ it is now widely used to investigate pain perception and autonomic responses.^20^^,^^21^ The procedure activates peripheral nociceptors and central pain pathways, along with broad systemic responses, including sympathetic activation and cardiovascular changes^20^^,^^22^ These responses may reflect not only nociception but also general stress reactions, autonomic, and cardiovascular responses, which must be considered when interpreting metabolomic data. Throughout this study, CPT is therefore treated as a standardized proxy for acute nociceptive stimulation embedded within a broader sympathetic stress response.

Breath analysis allows real-time, non-invasive monitoring of rapid metabolic changes in response to stressors like the CPT.^23^ Volatile metabolites can reflect phenotype with high temporal precision^24^^,^^25^ and may serve as biomarkers for personalized medicine applications, including pain.^26^^,^^27^^,^^28^^,^^29^ Moreover, breath analysis is particularly suitable for vulnerable patients, and we have previously applied it in diverse populations, including preterm infants and sedated individuals (see for example:^30^).

In this proof-of-concept study, we investigated whether real-time breath metabolomics can non-invasively detect physiological responses to acute pain. We exposed two independent cohorts to a standardized CPT protocol and measured exhaled breath using real-time high-resolution mass spectrometry. Our primary aim was to determine whether cold-induced pain elicits a reproducible metabolic signature in breath. Secondary objectives were to (1) interpret CPT-induced metabolic shifts, (2) validate findings across two geographically distinct laboratories, and (3) assess the feasibility of breath metabolomics as an observer-independent tool for real-time monitoring of pain-related physiological responses.

## Results

### Study design

**Figure 1:**
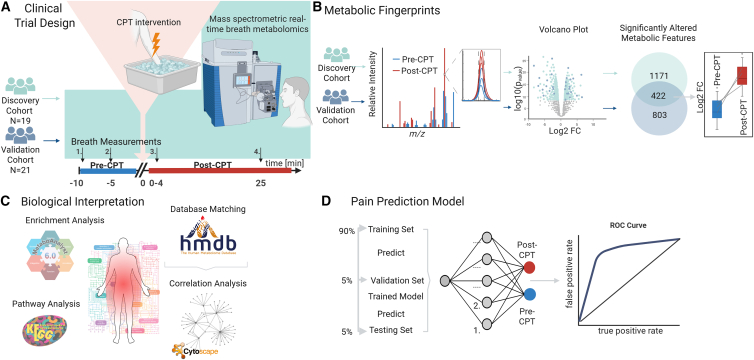
Flow chart of the overall study design and data analysis pipeline to unveil CPT-induced metabolic shifts (A) *N* = 40 participants from two centers (i.e., discovery cohort *n* = 19, and validation cohort *n* = 21) were involved in this study. They underwent the same CPT intervention and subsequent breath metabolic fingerprinting procedures. Breath specimens were subjected to real-time mass spectrometry-based metabolomics analysis before and after pain induction. (B) It followed a univariate and multivariate analysis pipeline to identify significant changes in exhaled metabolites. (C) Correlation and pathway analysis were then used to evaluate the similarities in response to CPT between both cohorts and for biological interpretation. (D) Neural network models were developed to ultimately predict whether the exhaled metabolic signature corresponded to CPT/no-CPT intervention.

The designation of “discovery” and “validation” cohorts reflects the chronological order of experimentation rather than population characteristics. The Swiss cohort was analyzed first, and the Chinese cohort was subsequently recruited to independently replicate the findings using identical instrumentation and protocols. A total of *n* = 20 healthy participants were enrolled in the University Children’s Hospital Basel in Switzerland (i.e., discovery cohort). One participant withdrew the consent retrospectively, hence *n* = 19 participants’ data were analyzed. A validation cohort of *n* = 21 healthy participants was recruited within Jinan University in China (i.e., validation cohort; Figure 1). The demographic characteristics of the discovery and validation cohorts were compared. The median age of participants in the discovery cohort was 26.0 years (interquartile range [IQR]: 5.5 years), while the median age in the validation cohort was 24.0 years (IQR: 5.0 years). A Mann-Whitney U test revealed no significant difference in age distribution between the two cohorts (U = 187.0, *p* = 0.744). Regarding gender distribution, the discovery cohort included 7 males and 12 females, while the validation cohort comprised 12 males and 9 females. A chi-square test found no significant difference in gender distribution between the cohorts (χ^2^ = 0.935, *p* = 0.334). The *N* = 40 participants of both sites completed four exhalation measurements, two before- (−15 and −5 min) and two after-CPT (+0 and +25 min) intervention. A multifaceted univariate and multivariate data analysis pipeline was deployed to identify altered metabolites/metabolic pathways because of CPT-induced pain, as well as to predict whether the breath mass spectral fingerprint corresponds to a pre- or post-CPT sample.

### Conserved metabolic signatures and network topologies following CPT

**Figure 2:**
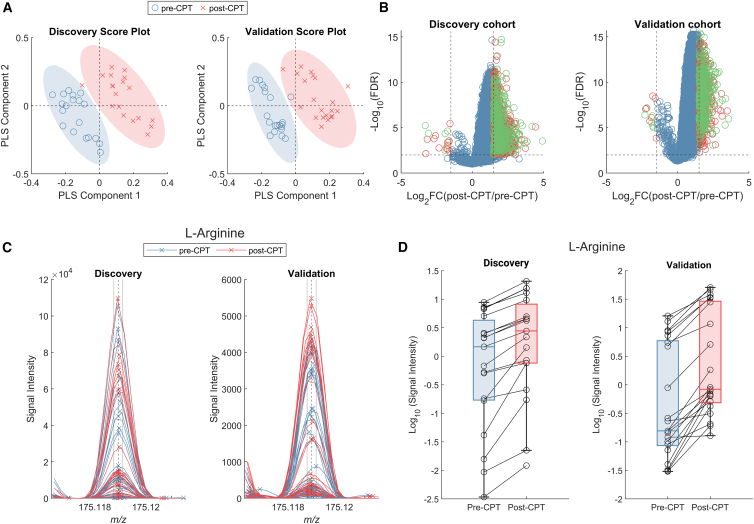
Concentrations of exhaled metabolites are increased upon CPT intervention (A) Partial Least Squares (PLS) score plots for both discovery and validation datasets show clear separation between pre-CPT (blue circles) and post-CPT (red crosses) metabolic profiles. This clustering suggests significant metabolic shifts induced by the CPT intervention, with consistent patterns across both datasets, supporting the robustness of the observed changes. (B) Volcano plot analysis performed on after-vs. post-CPT of all 5,058 metabolic features for the discovery and validation datasets. P-values were determined by a two-sided paired *t* test followed by adjustment for multiple comparison. Differentially regulated metabolic features detected in just one dataset are indicated by red dots. Green dots represent overlapping features between discovery and validation cohorts. (C) Representative example of an overlapping mass spectral identified by the volcano plot analysis, which was mapped to arginine. Each spectrum corresponds to one participant, whereas the dashed line indicates the theoretical *m/z* of protonated arginine ±1 ppm (solid vertical lines). (D) Signal intensity (Log10(AUC), see STAR Methods) of the same feature depicted as a boxplot overlaid with points, whereas every line represents an individual participant connecting pre-(blue) and post-CPT (red) boxplots. Discovery: mean Log_2_FC = 1.90; FDR <0.001 and validation: mean Log_2_FC = 1.58; FDR <0.001. Boxplots display the median (center line) and interquartile range (IQR; box limits). Individual data points represent single participants, with lines connecting pre- and post-CPT measurements for each participant.

The pain threshold, operationalized as the time participants could tolerate the cold-water stimulus, varied substantially across individuals, as reported in literature.^18^^,^^31^ The median (interquartile range) withstanding the hand in iced water was 65.93 (39.73–240) seconds and 35 (23–46) seconds for the discovery and validation cohort, respectively. The observed difference in tolerance times may reflect contextual factors such as reimbursement policies, environmental conditions, or site-specific experimental context. These factors were not systematically assessed and should be interpreted cautiously. Importantly, the study design did not aim to fine-tune pain intensity or exposure duration. Breath sampling was triggered immediately after hand withdrawal, and all analyses were based on a binary pre-versus post-CPT comparison, rendering absolute CPT duration secondary to pain occurrence. Such intervention resulted in a significant (*p* < 0.001) increase in blood pressure (Figure S1). A total of 5,058 mass spectral features were detected in both cohorts, of which 1,377 could be mapped to at least one metabolite from the Human Metabolome Database^32^ (Data S1). Whether CPT intervention was accompanied by a sizable change in the overall exhaled metabolic profile was initially assessed by partial least squares-discriminant analysis (PLS-DA). A 10-fold cross-validated analysis revealed a robust classification performance for both cohorts, as assessed by the accuracy (discovery: 0.97; validation: 0.93) and discriminant Q^2^^33^(discovery: 0.74; validation: 0.84). Figure 2A shows the resulting score plot based on SESI-HRMS breath mass spectra from the participants pre- and post-CPT. A clear shift of the data points upon the intervention is observed in both the discovery and validation cohorts, suggesting that the CPT elicited a consistent shift in the exhaled metabolic profile. To gain further insights into the strength and significance of such changes at the molecular level, we conducted a volcano plot analysis (Figure 2B). The figure indeed displays a striking change in the metabolic profile after CPT intervention, whereby the signal intensity of most of the features was increased upon intervention, as evidenced by the asymmetric volcano plot. We compared the area under the curve (AUC) of both measurements before CPT intervention against the AUC of both measurements after CPT intervention (i.e., measurements 1 + 2 versus 3 + 4). This approach integrates all four timepoints and accounts for potential variability between the two pre-intervention measurements. A total of 1,153 features were found to be significantly upregulated (Log_2_FC ≥ 1.5 & FDR ≤0.01) in the discovery cohort. In contrast, just 18 features were significantly decreased (log_2_FC ≤ −1.5 & FDR ≤0.01) after CPT. The analysis of the validation cohort revealed a remarkably similar picture, which exhibited 790 upregulated features and 13 downregulated features, respectively. A total of 416 upregulated and 6 downregulated features were consistently observed in both cohorts, indicating a reproducible metabolic response to CPT across study sites. Furthermore, hypergeometric analysis confirmed that this degree of overlap far exceeds what would be expected by chance alone (p ≪ 0.001), underscoring the robustness of the identified metabolic response.

The mass spectra of an example feature mapped to arginine are shown in Figure 2C. It demonstrates a consistent and significant increase as a response to the CPT across all subjects for the discovery (mean Log_2_FC = 1.90; FDR <0.001) and validation (mean Log_2_FC = 1.58; FDR <0.001) cohorts (Figure 2D). Figure S2 shows additional examples demonstrating similar trends for creatine, threonine, and asparagine. The complete heatmap of all 422 significant metabolic features across the four measurement timepoints for both cohorts is provided in Figure S4.

**Figure 3:**
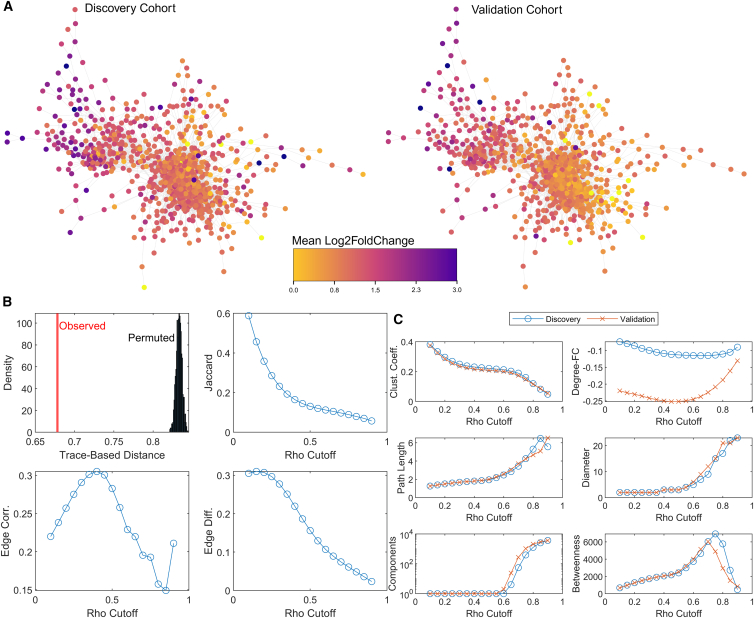
Cross-cohort correlation analysis of metabolite networks reveals consistent behavior following CPT-induced nociceptive stimuli (A) Correlation networks for discovery and validation cohorts at a ρ cutoff of 0.7. Node colors represent mean Log_2_ fold change (Log_2_FC), with higher fold changes clustering at the network periphery, indicating consistent intervention-driven effects across cohorts. (B) Cross-site similarity metrics reveal high structural similarity between networks, as indicated by significant Trace-Based Distance (0.678, *p* < 0.001) and eigenvalue correlation (0.992, *p* < 0.001). Metrics such as Jaccard Index and Edge Differences highlight increasing divergence at higher ρ cutoffs, reflecting site-specific variations in weaker metabolic connections. (C) Network topology analysis shows reduced clustering and increased fragmentation at higher ρ cutoffs, capturing the strongest intervention-driven pathways. Degree-FC correlations remain consistently negative, with hub nodes showing smaller fold changes. Metrics such as path length, diameter, and betweenness centrality highlight subtle differences in network topology while underscoring conserved metabolic responses across cohorts.

To gain further insights into whether the relationships across the metabolic features were conserved in the discovery and validation cohorts, we conducted a correlation analysis. Figure 3A shows the resulting correlation network (ρ cut-off >0.7 in both sites). The color coding of the nodes signifies the mean fold-change (FC) on Log_2_ scale (i.e., Log_2_FC). It becomes apparent that the features experiencing the strongest CPT-induced response tend to cluster together and are in the periphery of the network, whereas those with the lowest FC tend to correlate as well and are located at the core of the network. These correlation networks reflect the co-regulation patterns among metabolic features, where edges represent strong pairwise correlations. Visual inspection of the color distribution across both networks suggests a high degree of similarity, indicating that the data structure is preserved in the validation cohort. This conserved network architecture suggests that the underlying metabolic response to CPT is consistent across independent study populations.

Further analysis of discovery and validation networks across various ρ cutoffs reveals significant structural similarity between the two datasets (Figure 3B). The correlation matrix distance^34^ of 0.678 (*p*-value <0.001) indicates that the networks are highly similar. An eigenvalue correlation of 0.992 (*p*-value <0.001) confirms that the overall variance structures of the two networks are almost identical. However, the moderate correlation of correlations (0.187, *p*-value <0.001) and the Jaccard Index, which decreases from 0.59 at ρ = 0.1 to 0.06 at ρ = 0.90, suggest that differences in edge patterns and connectivity become more pronounced as weaker correlations are pruned. The weighted edge correlation, ranging from 0.22 to 0.31, reflects moderate consistency in edge weights, further underscoring a partial overlap in network connectivity. The edge difference metric, which decreases steadily from 0.305 at ρ = 0.1 to 0.023 at ρ = 0.9, highlights the reduction in the magnitude of edge weight differences between the two networks as weaker correlations are filtered out. This indicates a progressive alignment in the remaining stronger connections. Degree-FC correlation is consistently negative, becoming more pronounced at higher ρ cutoffs, with values ranging from −0.074 (*p*-value <0.001) to −0.113 (*p*-value <0.001) in the discovery cohort and from −0.220 (*p*-value <0.001) to −0.250 (*p*-value <0.001) in the validation cohort. This suggests that higher-degree nodes tend to exhibit smaller fold changes, reinforcing the role of hub nodes in maintaining network stability despite local differences. Both networks remain fully connected up to a ρ cutoff of 0.6, beyond which the number of connected components increases rapidly, indicating network fragmentation. The diameter of the networks remains stable at lower ρ values, with a value of 2, but begins to increase as the networks become sparser, reflecting reduced global connectivity. Metrics such as the clustering coefficient, which decreases from 0.38 to 0.05, and the average path length, which increases from 1.27 to 5.56, remain stable at lower ρ cutoffs but diverge as ρ increases. This divergence suggests that local connectivity and efficiency diminish as weaker edges are pruned, further contributing to network sparsity. Betweenness centrality shows a moderate decrease, from 679 to 480, as ρ increases, suggesting a shift in the importance of key nodes in maintaining overall connectivity. This reflects changes in the central landscape of the networks as they become fragmented. Overall, while local connectivity and specific edge patterns vary significantly with increasing ρ cutoffs, the global structural similarity between discovery and validation networks is consistent with the hypothesis that the networks may be underpinned by similar underlying metabolic processes.

### CPT-induced altered metabolic pathways

**Figure 4:**
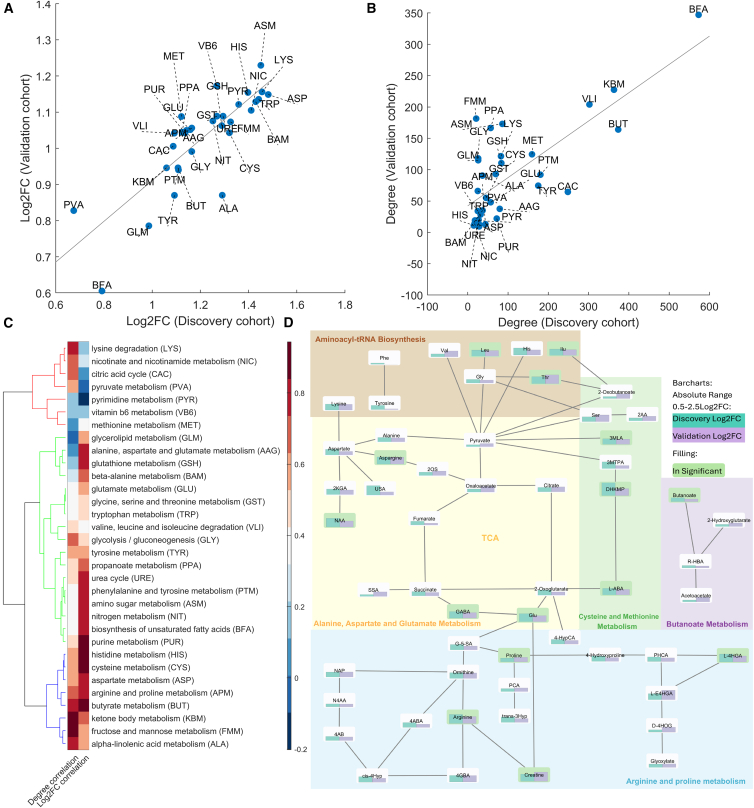
Pathway-based degree and enrichment analysis reveals a consistent multifaceted metabolic cascade in both cohorts (A) Comparison of median Log_2_ fold changes (Log_2_FC) between discovery and validation cohorts across metabolic pathways shows strong agreement. (B) Degree metrics, reflecting network connectivity, also exhibit high concordance between cohorts. (C) Hierarchical clustering of pathways based on intra-pathway Log_2_FC and degree correlations highlights consistent perturbations in pathways such as alpha-linolenic acid metabolism (ALA), fructose and mannose metabolism (FMM), ketone body metabolism (KBM), butyrate metabolism (BUT), arginine and proline metabolism (APM), cysteine metabolism (CYS) and histidine metabolism (HIS). These pathways underscore the body’s metabolic response to CPT, focusing on energy homeostasis and biosynthesis. (D) A schematic representation of enriched pathways, derived from mummichog analysis, connects Aminoacyl-tRNA biosynthesis, Cysteine and methionine metabolism, Butanoate metabolism, Alanine, aspartate, and glutamate metabolism, and Arginine and proline metabolism. These pathways integrate fatty acid, carbohydrate, and amino acid metabolism to reflect the primary metabolic shifts following CPT.

The univariate, multivariate, and correlation network data analysis approaches presented above suggest a stark and rapid metabolic perturbation in response to the CPT intervention, consistent across both cohorts. To generate further hypotheses regarding the specific altered regions of the human metabolome, we conducted a pathway-based degree analysis. A total of 55 metabolic pathways were associated with the 5,058 mass spectral features detected in both cohorts (Data S1). To increase the robustness of the analysis, we further considered only those pathways represented by at least five mass spectral peaks, reducing the list to 32 pathways. Figures 4A and 4B show a strong agreement in median Log_2_FC and degree between the discovery and validation cohorts. Further insights into the intra-pathway correlation for the Log_2_FC and the degree were obtained by conducting a hierarchical cluster analysis visible in Figure 4C. This analysis revealed that the most consistent behavior between the discovery and validation cohorts regarding both Log_2_FC and the degree, was found for alpha-linolenic acid metabolism (ALA), fructose and mannose metabolism (FMM), ketone body metabolism (KBM), butyrate metabolism (BUT), arginine and proline metabolism (APM), aspartate metabolism (ASP), cysteine metabolism (CYS), and histidine metabolism (HIS). These pathways collectively reflect the body’s ability to convert and utilize fatty acids, carbohydrates, and amino acids for energy homeostasis and biosynthetic functions.^35^^,^^36^

To complete the biological interpretation of CPT-driven metabolic shifts, we conducted an enrichment analysis using the mummichog algorithm.^37^ We defined the significant feature list as those features that were significantly altered in both study sites (i.e., 8% of the total feature list; 416/upregulated/6 downregulated; Data S1). The top five pathways (p_gamma_ < 0.1) included Aminoacyl-tRNA biosynthesis, Cysteine and methionine metabolism, Butanoate metabolism, Alanine, aspartate and glutamate metabolism, and Arginine and proline metabolism (Figure S3), comprising a total of 58 compounds mapped to these pathways (Data S2). Figure 4D shows a schematic representation of how the main identified pathways are interconnected, providing an overview of the principal metabolic alterations driven by CPT.

### Neural networks predict whether a breath mass spectral fingerprint is associated with CPT

**Figure 5:**
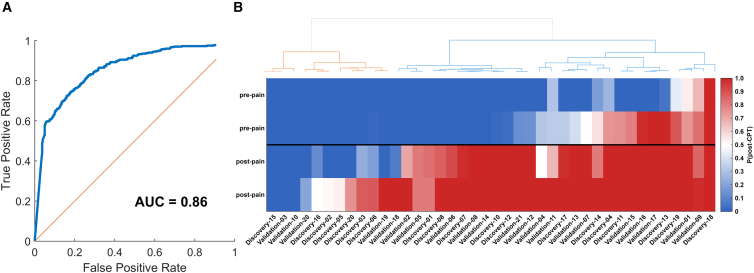
Neural network-based prediction of CPT intervention status using breath mass spectral fingerprints (A) Receiver operating characteristic (ROC) curve for the neural network model trained to classify pre- and post-CPT breath fingerprints. The model achieved an area under the curve (AUC) of 0.86 and an overall accuracy of 78%. (B) Heatmap of predicted classification probabilities for each of the four breath measurements per subject across the Discovery and Validation cohorts. Each cell represents the mean probability averaged across cross-validation folds that a given breath sample is classified as post-CPT, ranging from 0 (confidently classified as pre-CPT) to 1 (confidently classified as post-CPT). The decision boundary is 0.5: values above this threshold are predicted as post-CPT, values below as pre-CPT. Subjects (columns) are ordered by hierarchical clustering (Ward linkage) of their individual probability profiles. Hierarchical clustering revealed three distinct subject clusters: (1) a pre-CPT cluster consisting of samples primarily classified with low probabilities of CPT exposure, (2) a post-CPT cluster with high probabilities of correct classification as CPT-induced, and (3) a mixed cluster where some pre-CPT samples were incorrectly classified as CPT-induced, potentially reflecting a heightened baseline metabolic state or anticipatory response prior to the intervention.

The data presented above suggests rapid and stark metabolic changes taking place in response to CPT intervention. Therefore, we trained a neural network model to test the hypothesis that a breath-based mass spectral fingerprint can distinguish between pre- and post-CPT states. The resulting ROC curve (Figure 5A) summarizes the model’s performance, with an AUC of 0.856 and an overall accuracy of 78%. When analyzing predictions at an individual level (Figure 5B), we observed that most participants were correctly classified: the first two measurements (i.e., pre-CPT) were typically assigned a low probability of belonging to the CPT-induced metabolic state, while the last two measurements (i.e., post-CPT) were assigned a high probability. However, some individuals were systematically misclassified; for example, the second measurement was sometimes labeled as CPT-induced, and the third as pre-CPT. Hierarchical clustering analysis revealed three main clusters. The first cluster predominantly consisted of samples classified pre-CPT, suggesting that the metabolic response to CPT was either delayed or less pronounced. The second cluster comprised post-CPT samples that were consistently classified as CPT-exposed, reflecting a robust metabolic shift following the intervention. The third cluster contained a mix of pre- and post-CPT samples, with some pre-CPT samples misclassified as post-CPT. Overall, the correct classification rates for the first, second, third, and fourth breath measurements were 92.5%, 70%, 70%, and 87.5%, respectively. Misclassifications were observed in both the discovery and validation cohorts.

## Discussion

Our findings establish proof-of-concept for a real-time, observer-independent approach to detect short-term metabolic responses to a standardized nociceptive stimulus that simultaneously activates sympathetic and cardiovascular pathways using exhaled breath. While not directly compared to standard pain scores (numeric rating scale, NRS, visual analog scale, VAS, verbal rating scale, VRS), this study demonstrates the potential of real-time breath metabolomics via SESI-HRMS and machine learning to detect acute metabolic shifts triggered by a standardized nociceptive stimulus. Further validation is needed in diverse clinical populations and under real-world conditions. These preliminary results provide a basis for future testing in clinical contexts where self-report is not possible.

Breath-based assessment of metabolic response profiles may eventually enable personalized treatment strategies, although this potential remains to be demonstrated in clinical settings. Pain is a complex, multifactorial phenomenon that varies with individual, disease-related, and contextual factors. Existing research into physiological proxies of nociception, such as SC, has yielded mixed results. For example, Hullett et al. demonstrated that SC could reliably predict the absence of moderate to severe pain in postoperative pediatric patients.^15^ Dalal et al. found that peak SC values may indicate unmitigated nociceptive responses in infants.^16^ In contrast, Solana et al.^38^ concluded that SC was not more sensitive or faster than clinical scales for pain assessment in critically ill children. Hu et al. also reported inconsistent validity evidence for SC in pain assessment, noting significant correlations with unidimensional behavioral pain scales but not with multidimensional measurements.^17^

Characterizing a sensory and emotional experience such as pain at a molecular level is inherently challenging. The physiological response to noxious stimuli involves activation of the sympathetic nervous system (SNS), increasing sympathetic tone across multiple organ systems. These responses mobilize energy resources and prioritize survival-critical functions. In this study, we used the CPT as a standardized proxy for acute nociceptive stimulation and assessed associated metabolic changes through a non-invasive, breath-based approach. Our results suggest that CPT triggers, within 15 min, a marked upregulation of approximately 400 mass spectral features in exhaled breath, with a high degree of conservation across both European (discovery) and Chinese (validation) cohorts. These metabolic profiles may reflect individual physiological responses to acute sympathetic activation and could potentially support future personalized treatment strategies.

Subsequent pathway enrichment and degree analysis identified coordinated changes in aminoacyl-tRNA biosynthesis, cysteine/methionine metabolism, butanoate metabolism, alanine/aspartate/glutamate metabolism, and arginine/proline metabolism. Enhanced aminoacyl-tRNA biosynthesis may reflect stress-related protein synthesis demands.^39^ Upregulation of cysteine and methionine metabolism may reflect enhanced antioxidant defenses and redox control, potentially linked to catecholamine metabolism.^40^ Butanoate metabolism may contribute to alternative energy substrates and modulate inflammation.^41^ Alterations in alanine, aspartate, and glutamate metabolism—particularly glutamate’s central role in nociceptive neurotransmission—could signal involvement of central pathways responsive to the CPT stimulus.^42^ Finally, the arginine–proline axis, which regulates nitric oxide (NO) production^43^ and tissue remodeling, may act in synergy with sympathetic activation to fine-tune vascular tone, aligning with the observed blood pressure increase following CPT.

This high-level metabolic shift aligns with prior work showing that CPT induces changes at both the transcriptomic and metabolomic levels, reflecting SNS activation in response to stress and nociceptive stimuli.^28^^,^^44^ For example, arginine has previously been shown to correlate with gene expression changes following CPT, consistent with our observation of elevated exhaled arginine levels. Arginine is a key amino acid in NO production and contributes to vascular regulation and energy mobilization —mechanisms that support the body’s adaptation to acute physiological stress.^45^^,^^46^ Glutamic acid was also elevated. As an excitatory neurotransmitter, glutamate may play a role in central stress integration and nociceptive signal amplification, potentially contributing to blood pressure regulation in this context.^47^^,^^48^^,^^49^ Gamma-aminobutyric acid (GABA), typically inhibitory, also showed elevated levels. Dysregulation in the balance between excitatory and inhibitory neurotransmitters, including GABA, may be linked to increased SNS excitability, pain perception, and blood pressure. Creatine was also upregulated, consistent with its role in energy metabolism during SNS activation.^50^

Interestingly, our findings parallel those from recent meta-analyses in chronic pain research, which report alterations in amino acid metabolism (e.g., glutamine, serine, and phenylalanine) and macromolecular intermediates.^51^ In the context of cold-induced pain, a recent meta-analysis of metabolomics studies^52^ found that elevated glutamate levels —either systemically or locally —have been observed in a range of conditions, including fibromyalgia, complex regional pain syndrome, trapezius myalgia, and mixed musculoskeletal pain conditions. Glutamate signaling is widely recognized to play a central role in both central and peripheral sensitization mechanisms.^53^^,^^54^^,^^55^ Although the CPT-induced pain in our study does not model chronic pain, the involvement of glutamate-related pathways appears to be a consistent finding across diverse nociceptive contexts. Moreover, chronic pain patients often exhibit heightened sensitivity to decreasing temperatures,^56^ suggesting a broader relevance of cold-induced sensory mechanisms. Metabolites tied to glutamate signaling may indicate nociceptive processing in acute and chronic conditions.

This is further supported by the known role of cold-sensitive TRP channels. Specifically, TRPA1 activation—triggered by noxious cold and chemical irritants—has been shown to enhance glutamate release onto the nucleus tractus solitarii.^57^^,^^58^^,^^59^ This mechanism highlights a possible link between cold-induced sensory input and glutamatergic neurotransmission. Altogether, our data underscores the importance of amino acid and carbohydrate metabolism for meeting immediate energy demands, alongside lipid and redox pathways that support sustained adaptation. This dynamic prioritization of metabolic routes reflects the body’s metabolic flexibility in responding to acute stress.^60^^,^^61^ Beyond advancing our understanding of these molecular processes, our neural network predictions (Figure 5) suggest that rapid shifts in exhaled metabolic patterns may one day support objective physiological monitoring in clinical contexts. However, further validation is needed before such breath-based methods can be translated to diverse pain or stress-related conditions.

A key strength of our study lies in the inclusion of a geographically and analytically independent validation cohort, which exhibited a remarkably similar metabolic response to CPT. Highly standardized sampling protocols and instrumental conditions across both laboratories minimized technical variability and ensured high mass accuracy.^62^^,^^63^ Additionally, several core metabolite classes central to this work—such as amino acids^64^ and intermediates of the citric acid cycle^65^ —have previously been validated in exhaled breath, lending further credibility to the observed spectral patterns. In summary, we demonstrate that exposure to the CPT induces a rapid physiological response, including a pronounced metabolic shift detectable within minutes. This was observed using real-time high-resolution mass spectrometry to interrogate the exhaled metabolome. The resulting changes reflect the complex interplay between SNS activation, energy mobilization, vascular regulation, and nociceptive signaling. The altered pathways involve key roles in neurotransmission, circulatory control, and metabolic adaptation, contributing to the body’s acute stress response. Taken together, these interlinked mechanisms appear to optimize energy use, modulate vasoactive processes, and support neural signaling—consistent with the hemodynamic elevation and sensory activation associated with CPT. Importantly, we showed that these exhaled metabolic fingerprints can be used to non-invasively and in real time estimate the likelihood of CPT exposure through machine learning-based classification. Replication in two independent cohorts supports the robustness and generalizability of the metabolic signature. These findings highlight the potential of exhaled metabolomics as a powerful, observer-independent tool to support real-time assessment of physiological responses relevant to pain monitoring, particularly in vulnerable or non-communicative patient populations. To translate these findings into clinical practice, future studies should test this approach in patient populations with varying pain conditions and communication barriers. Integration with clinical scoring systems and comparison across different nociceptive models will be critical for clinical validation.

### Limitations of the study

It is important to acknowledge several limitations of this study. First, the inclusion of a relatively small number of study sites and participants, typical in early-phase metabolomics research, may have reduced statistical power despite the high dimensionality of the data. Additionally, although sex-specific metabolic differences are plausible, the present sample size precluded statistically meaningful sex-stratified analyses. Future studies with larger cohorts will be required to address sex-dependent effects. Second, the identification of metabolites in exhaled breath was based on accurate mass matching within a narrow ±1 ppm window using the Human Metabolome Database. While this enables putative annotation, it lacks the structural specificity offered by orthogonal techniques such as tandem mass spectrometry or nuclear magnetic resonance spectroscopy. Consequently, the biochemical interpretation of these features should be approached with caution until further validated through confirmatory analyses. Third, CPT elicits a combination of physiological responses, including nociceptive, thermoregulatory, cardiovascular, and stress-related processes, making it difficult to isolate the metabolic effects of pain per se. However, this overlap also reflects the multifactorial nature of pain in real-world settings and highlights the potential value of assessing pain within broader physiological frameworks. Fourth, although several metabolic pathways and features highlighted in this study have been associated with nociception or pain processing in the literature, many supporting studies are limited in size and design. The specific involvement of these molecules in pain signaling, sympathetic activation, and cold transduction remains to be fully characterized. Fifth, pain-related metabolomics is still an emerging field. While numerous candidate biomarkers are currently being reported, it remains to be seen which findings will prove robust and reproducible across cohorts and clinical conditions.

## Resource availability

### Lead contact

Requests for further information and resources should be directed to and will be fulfilled by the lead contact, Pablo Sinues (pablo.sinues@unibas.ch).

### Materials availability

This study did not generate new unique reagents, plasmids, cell lines, animal models, or other physical materials. No materials transfer agreements are required.

### Data and code availability

• Raw breath metabolomics data and associated metadata have been deposited at Mendeley Data as Mendeley Data: https://doi.org/10.17632/9g3nthjf2z.2 and are publicly available as of the date of publication. The dataset includes raw SESI-HRMS files, Exhalion files, subject metadata, measurement conditions, instrumental parameters, and CPT response times for both the discovery and validation cohorts.
• This paper does not report original code. All data processing and analysis steps are fully described in the STAR Methods and supplemental information.
• Any additional information required to reanalyze the data reported in this paper is available from the lead contact upon request.

## Acknowledgments

Funding Sources: 10.13039/501100011318Fondation Botnar (Switzerland) no. 320030_173168 (PS), 10.13039/501100001711Swiss National Science Foundation (10.13039/501100001711SNSF, 10.13039/501100001711Schweizerischer Nationalfonds zur Förderung der Wissenschaftlichen Forschung) PCEGP3-181300/501100001711-173168 (PS), Guangdong Major Project of Basic and Applied Basic Research (no. 2023B0303000013) (XL), Guangdong Provincial International Science and Technology Cooperation Project (no. 2022A0505050044) (XL), 10.13039/501100001809National Natural Science Foundation of China (no. 22122603) (XL).

## Author contributions

Conceptualization: M.R. methodology: P.S., K.D.S., and M.R. investigation: M.R., D.S., S.B., L.P., Y.S., Z.T., Xi.L., and X.L. formal analysis: P.S., M.R., K.D.S., and P.C.C. visualization: M.R., P.S., and K.D.S. supervision: P.S. writing – – original draft: M.R. and P.S. writing – review and editing: P.S., M.R., U.F., P.C.C., X.L., J.G., K.D.S., D.S., and S.B.

## Declaration of interests

PS is co-founder and board member of Deep Breath Intelligence (DBI) AG, a company that provides services in the field of breath analysis. Kapil Dev Singh is partially employed by the same company. UKBB is a shareholder of said company. All other authors declare they have no competing interests.

## Declaration of generative AI and AI-assisted technologies in the writing process

During the preparation of this work the authors used ChatGPT (GPT4 to 5, OpenAI) to improve readability and language. After using these tools, the authors reviewed and edited the content as needed and take full responsibility for the content of the publication.

## STAR★Methods

### Key resources table

**Table:** 

REAGENT or RESOURCE	SOURCE	IDENTIFIER
**Biological samples**		

Healthy Adult Real-time Breath Samples	University Childrens Hospital Basel, Switzerland	–
Healthy Adult Real-time Breath Samples	Jinan University, Guangzhou, P.R. China	–

**Deposited data**		

RAW Breath Data	Mendeley Data	https://doi.org/10.17632/9g3nthjf2z.2
Metadata both Cohorts	Mendeley Data	https://doi.org/10.17632/9g3nthjf2z.2

**Software and algorithms**		

Thermo Exactive Plus Tune software (Discovery Cohort)	Thermo Fisher Scientific	Version 5.0.0.38
Thermo Exactive Plus Tune software (Validation Cohort)	Thermo Fisher Scientific	Version 2.8.1.2806
RawFileReader	Thermo Fisher Scientific	Version 5.0.0.38
Cytoscape	https://cytoscape.org/	Version 3.10.2
MetaboAnalystR	https://www.metaboanalyst.ca/	Version 4.0.0
Python	https://www.python.org/	Version 3.12.3
MATLAB	MathWorks Inc., USA	R2023a–R2024a

**Other**		

KEGG Homo Sapiens (humans) database hsa		https://www.kegg.jp/
Seven Golden Rules for heuristic filtering of molecular formulas obtained by accurate mass spectrometry	Kind, T., Fiehn, O. et al.	https://doi.org/10.1186/1471-2105-8-105
Human Metabolome Database	https://www.hmdb.ca/	Version 5.0

### Experimental model and study participant details

#### Human participants

This study enrolled healthy adult volunteers across two independent cohorts. The Discovery Cohort comprised 19 participants (7 male, 12 female) of White European ancestry (mean age 28.8 years, SD 9.8, range 18.7–55.4 years), recruited at a site in Basel, Switzerland. The Validation Cohort comprised 21 participants (12 male, 9 female) of Chinese ancestry (mean age 25.9 years, SD 2.8, range 22.1–31.2 years), recruited in GuangZhou, China. Participants were recruited via word of mouth and advertisements.

Participants were required to be healthy adults. Exclusion criteria applied equally to both cohorts and included: regular use of medications that may affect pain perception (analgesics, antihistamines, calcium and potassium channel blockers, psychoactive substances, or narcotics); pregnancy; daily smoking (per WHO definition); neuropathy; chronic or persistent pain lasting more than three months; neuromuscular or psychiatric disorders; known or suspected cardiac, renal, or hepatic disease; hypertension (systolic >130 mmHg or diastolic >80 mmHg); history of Raynaud’s phenomenon; history of fainting or seizures; history of frostbite; and current psychological or psychiatric treatment. All participants provided written informed consent prior to enrollment.

**Table:** 

Sex	Age (y)	Sex	Age (y)
Discovery Cohort		Validation Cohort	
White European		Chinese	
Male	21.7	Male	31.0
Female	27.9	Male	24.2
Female	26.4	Female	24.0
Male	40.1	Female	30.7
Male	22.4	Female	29.1
Female	22.8	Male	23.9
Female	55.4	Female	25.3
Female	28.5	Male	23.4
Male	20.5	Male	27.3
Male	27.5	Female	23.9
Male	27.0	Male	31.2
Female	22.4	Male	23.9
Female	22.9	Male	23.8
Female	24.7	Male	26.7
Female	27.1	Male	24.4
Male	18.7	Male	23.3
Female	23.5	Female	24.6
Female	47.7	Female	28.7
Female	39.7	Female	22.1
	–	Female	24.2
	–	Male	28.5

Table about Information on study participants.

##### Influence of sex on study results

The influence of sex on study outcomes was not formally assessed, as the overall sample size was limited and further subdivision by sex would result in groups too small for meaningful statistical inference. This represents a limitation of the current study, and future work with larger samples should examine potential sex-related differences in the outcomes reported here.

#### Ethics approval

Consent to participate including consent for publication: All participants provided written consent in accordance with the respective national laws.

Clinical trial registrations and ethical approvals for this study are detailed below.

Description: Swiss national clinical trial registry entry for the Discovery Cohort (Basel, Switzerland):

https://www.humanforschung-schweiz.ch/en/trial-search/study-detail/55364#query=SNCTP000004536.

Description: ClinicalTrials.gov registration for the Discovery Cohort: https://clinicaltrials.gov/study/NCT04956718.

**Table:** 

Name	ID	Site
BASEC ID:	2021–01132	CH
WHO(ICTRP) ID:	NCT04956718	CH
Kofam ID:	SNCTP000004536	CH
DKF CTU Basel ID:	ks21Sinues	CH
JNUKY	2023-0059.	CN

Table listingPre-Registrations and ethical committee approvals.

### Method details

#### Experimental design: CPT as nociceptive stimulus

A standardized CPT was used to induce acute physiological and nociceptive responses.^66^^,^^67^^,^^68^ During the trial, each participant underwent four exhalation measurements (Figure 1): Two baseline sets before the CPT, one immediately after the hand withdrawal from the cold water, and a subsequent final washout measurement 15 minutes after the third measurement. Participants were instructed to submerge their open, relaxed right hand—including approximately 3 cm of the wrist—into a container filled with ∼13 L of ice-cold water and crushed ice. The water was stirred manually, and the temperature was continuously monitored using a laboratory thermometer to maintain 2°C ± 1°C. Participants were asked to keep their hand submerged for as long as they could tolerate the discomfort. A maximum duration of 4 minutes was imposed; participants who reached this cutoff were instructed to remove their hand.

All participants received study information and consent materials by email at least 24 hours prior to the experimental session. Two versions of the information sheet were provided: one in simplified language and one in more detailed scientific language. Upon arrival at the study site, the entire procedure was reviewed again, and any participant questions were addressed prior to consent. Written informed consent and general consent were obtained from all participants before data collection began. Prior to the start of the project, the study protocol was approved by the appropriate ethics committee (Swiss equivalent to Institutional Review Board, IRB), with the study endpoints and procedures registered accordingly.

Sample size was selected based on prior real-time breath metabolomics studies using within-subject repeated-measures designs. Each participant served as their own control with two pre- and two post-CPT measurements, substantially increasing statistical power for detecting intervention-related metabolic changes despite modest cohort sizes. Given the exploratory, proof-of-concept nature of the study, no formal *a priori* power calculation was performed.

#### Real-time breath metabolomics by high-resolution mass spectrometry

The breath metabolomics platform was based on a Secondary Electrospray Ionization-High Resolution Mass Spectrometry (SESI-HRMS) setup. Identical or harmonized settings were used across both study sites unless otherwise specified. The system consisted of a direct coupling between a high-resolution mass spectrometer (Q-Exactive Plus in the Swiss site and Q-Exactive in the Chinese site, Thermo Fisher Scientific, Germany) and a Super SESI ion source (Fossiliontech FIT, Spain).

A 20 μm capillary emitter (Fossiliontech FIT, Spain) was employed to generate an electrospray from a water solution containing 0.1% (v/v) formic acid (LiChrosolv, hypergrade for LC-MS, 1.59013.2500, Millipore). The pressure within the electrospray reservoir was maintained at 1.3 bar at the Swiss site and 0.8 bar at the Chinese site. Electrospray voltage was set to 2.8 kV for both positive and negative ionization modes.

The ionization chamber and sample line were maintained at temperatures of 90°C and 130°C, respectively. The Orbitrap capillary temperature was set to 275°C, the sheath gas flow rate to 60 arbitrary units (AU), and the S-lens RF level to 55.0 V. Mass spectra were acquired using Thermo Exactive Plus Tune software (5.0.0.38 for the Swiss site and 2.8.1.2806 for the Chinese site) operated in full scan mode with a resolving power of 140,000 at m/z 200. Scan parameters included a mass range of 70–1000 m/z, polarity switching between positive and negative modes, two micro-scans, an ACG target of 1e6, and a maximum injection time of 500 ms.

The MS was internally calibrated by enabling lock masses corresponding to typical background mass spectrometric contaminants.^69^^,^^70^ External calibration was performed weekly using a commercially available calibration solution (PierceTM Triple Quadrupole, extended mass range).^71^ Each day prior to any breath measurement, an external gas standard (α-terpinene at 100 ppb | Dalian Special Gases Co. Ltd, Dalian, China)^72^ was used to test the instrument’s sensitivity. Instrument performance was evaluated against historical data using Nelson control chart criteria,^73^ ensuring that the system operated within defined “in-control” limits. Only after passing this suitability check were participants invited to proceed with breath sampling. The procedure followed a previously standardized protocol.^62^^,^^74^ Briefly, each exhalation set consisted of six exhalations in positive ion mode and six exhalations in negative ion mode. Exhalations were guided by real-time CO_2_ monitoring and regulated by a mass flow controller. The duration of one exhalation set was approximately Seven minutes.

### Quantification and statistical analysis

#### Pre-processing of mass spectrometric data

Mass spectral data preprocessing followed our previously described and patented pipeline^75^^,^^76^: Acquired ∗.RAW files were preprocessed using the in-house developed SESI-HRMS Analysis Toolbox (version 5.2.0) implemented in MATLAB (R2023a–R2024a; MathWorks Inc., USA) and C#. Briefly, exhalation time windows were identified by isolating intervals in which CO_2_ concentrations exceeded 3%, corresponding to the end-tidal fraction (measured using the Exhalion capnograph). Average raw centroid and profile mass spectra from these intervals were computed using custom C# console applications developed with Thermo Fisher Scientific’s RawFileReader (version 5.0.0.38). Both centroid and profile spectra were then recalibrated to achieve mass accuracy within ±1 ppm. Apodization was applied to minimize satellite peak artifacts.^77^ The resulting centroid data were subsequently binned using MATLAB’s *ksdensity* function, also within a ±1 ppm tolerance window. This preprocessing pipeline yielded a final feature list consisting of 11,840 features in positive ion mode and 4,446 features in negative ion mode.

#### Post-processing of mass spectrometric data

To reduce data sparsity, all mass spectral features with ≥50% zero values across all samples were excluded. This filtering step reduced the feature set to 3,734 features in positive mode and 1,324 in negative mode, yielding a total of 5,058 features for downstream analysis. Remaining zero values were imputed using the regression on order statistics method.^78^

To quantify rapid changes in breath composition in response to the CPT intervention, the two pre-CPT and two post-CPT measurements were aggregated by calculating the area under the curve (AUC) between each pair of measurements. These AUCs were then normalized by the time interval between the respective measurements. For each feature, the Log_2_ fold change (Log_2_FC) between pre- and post-CPT AUCs was computed. Statistical significance was assessed using a paired *t* test. Features were considered significantly regulated if they met both of the following criteria: a false discovery rate (FDR) ≤ 0.01 and an absolute Log_2_FC ≥ 1.5.^79^

#### Biological interpretation: Database query, correlation, and enrichment analysis

Final spectral features were matched to molecular formulas using the following ion types: protonated ions [M + H]^+^, their ^13^C isotopologues [M(^13^C)+H]^+^, deprotonated ions [M–H]^-^, and their corresponding ^13^C isotopologues [M(^13^C)–H]^-^, applying a mass error tolerance of ±1 ppm. Candidate formulas were filtered using the “Seven Golden Rules for heuristic filtering of molecular formulas obtained by accurate mass spectrometry.”.^80^ Molecular formulas were subsequently mapped to the Human Metabolome Database (HMDB, version 5.0), using the “serum metabolites” dataset.^32^ HMDB identifiers, taxonomy classifications, pathway associations, and metabolite descriptors were retrieved for matched entries.

Correlation networks were constructed using Pearson correlation coefficients and visualized using Cytoscape (version 3.10.2).^81^ Feature pairs with ρ > 0.7 in both study sites (discovery and validation) were retained. Only node pairs meeting this correlation threshold consistently across both cohorts were selected for further analysis. These reproducible correlations were assembled into a tabular format representing strongly co-varying feature pairs.

Pathway enrichment analysis was performed using the mummichog algorithm as implemented in MetaboAnalystR (version 4.0.0), referencing the hsa_kegg database and adduct list and mass tolerance described above. In cases of overlapping peaks, a lower probability weight of 0.001 was assigned to the corresponding entries in dummy Ps vector (initialized to 1), allowing for down-weighting of ambiguous matches and improved distinction between unique and overlapping signals.

#### Prediction of CPT state using neural networks

We employed a multi-layer perceptron (MLP) neural network in a Python environment to classify pre-versus post-CPT breath mass spectra. Prior to modeling, profile-mode spectra were linearly interpolated and concatenated across positive and negative ion modes, resulting in a unified feature vector per breath sample. The final data matrix consisted of 160 samples (four samples per participant: two pre-CPT, two post-CPT), derived from 19 participants in the discovery cohort and 21 in the validation cohort. Dimensionality was reduced using principal component analysis (PCA), evaluating component sets of 48, 64, 96, 128, and 144 principal components. These served as inputs to the neural network. Each network architecture included an input layer corresponding to the number of PCA features, followed by two hidden layers. We evaluated hidden layer sizes of [32, 32], [48, 48], [64, 64], and [96, 96]. For model evaluation, participant-level stratified data splitting was applied: 90% training, 5% validation, and 5% testing. All four samples from a given participant were assigned to the same split (training, validation, or test) to avoid data leakage and classification bias. For each combination of PCA dimensionality and hidden layer size, this process was repeated 50 times with random partitions per fold. PCA was performed only on the training data, and the test samples were projected onto the same PCA space before prediction. Model performance was evaluated using the area under the receiver operating characteristic (ROC) curve (AUC). The best classification performance was achieved with 48 PCA components and a network architecture of [96, 96] hidden neurons. This configuration yielded an average AUC of 0.856 and an overall classification accuracy of 78%.

## Supplemental information

Document S1. Figures S1–S4

Data S1. Mass spectral feature annotations, mapped metabolites, and pathway associations for all 5,058 features detected across both cohorts

Data S2. Compounds mapped to the top five enriched metabolic pathways identified by mummichog analysis
