# Supplementary material for: Cold induced pain elicits reproducible breath metabolomic responses across geographically distinct populations
Source: iScience. 2026 Apr 22;29(6):115857. doi: 10.1016/j.isci.2026.115857 (PMC13156685; doi:10.1016/j.isci.2026.115857)
Supplement: Document S1. Figures S1–S4 [file mmc1.pdf]

iScience, Volume *29*

Supplemental information

Cold induced pain elicits reproducible breath metabolomic responses across geographically distinct populations

Mélina Richard, Kapil Dev Singh, Dilan Sezer, Sarah Buergler, Luana Palermo, Yannick Schulz, Zhifeng Tang, Xin Luo, Urs Frey, Philippe C. Cattin, Xue Li, Jens Gaab, and Pablo Sinues

This PDF file includes:

Supporting text
Figures S1 to S4

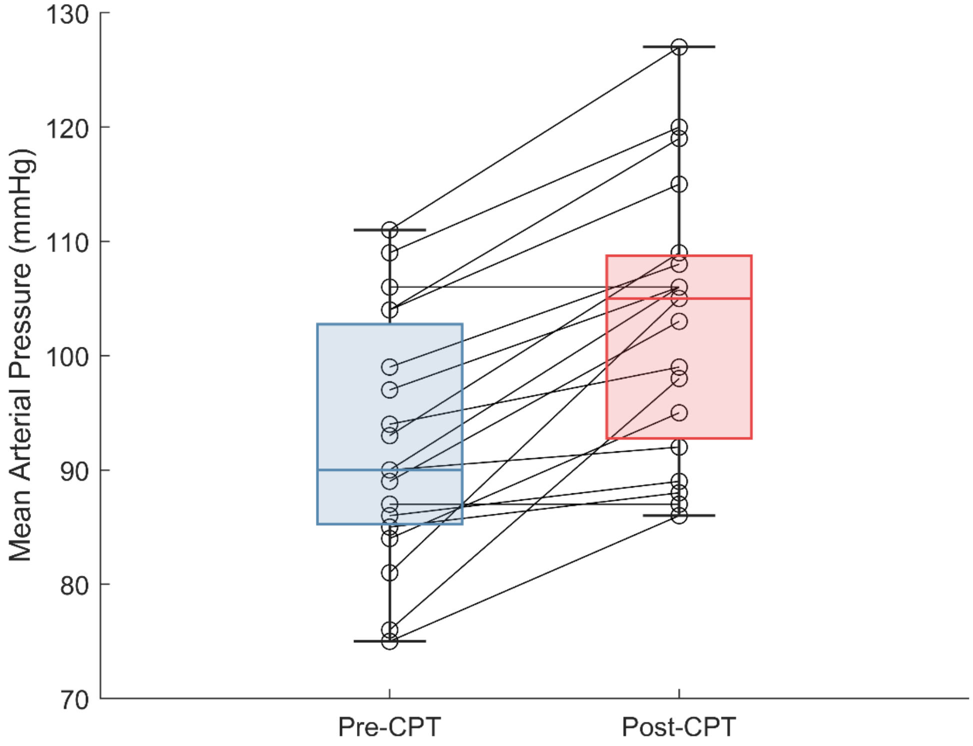

Fig. S1. Mean arterial pressure increases upon CPT intervention

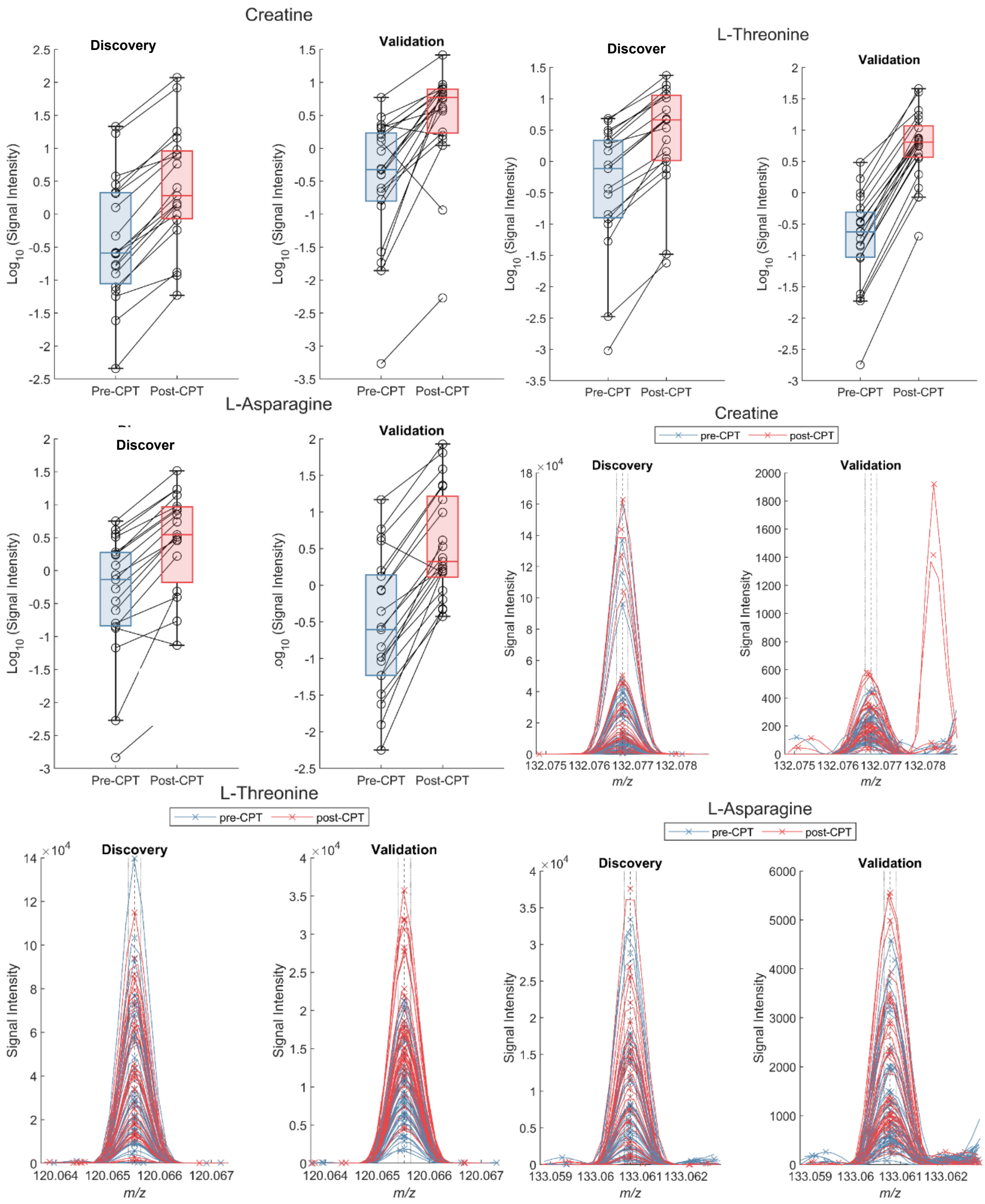

Fig. S2. Further examples of significantly increased exhaled metabolites

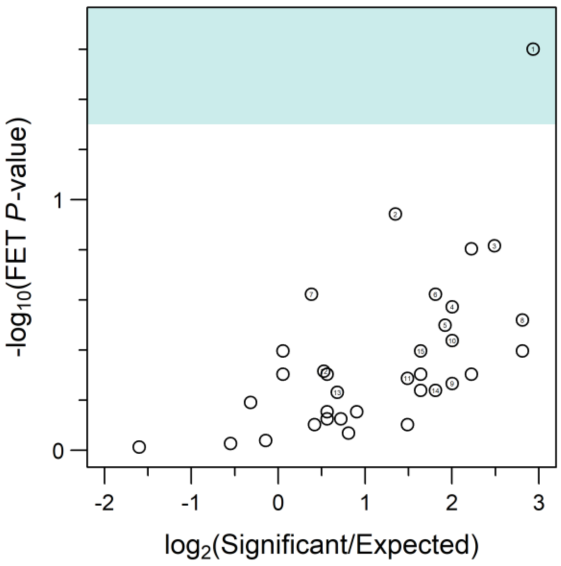

	Pathways	Size	Matches			*P*−values		
			Total	Significant	Expected	FET	EASE	Γ
1	Aminoacyl-tRNA biosynthesis	22	16	6	0.79	0.025	0.080	0.058
2	Cysteine and methionine metabolism	33	8	3	1.18	0.11	0.35	0.074
3	Butanoate metabolism	15	9	3	0.54	0.15	0.41	0.078
4	Alanine, aspartate and glutamate metabolism	28	17	4	1.00	0.27	0.50	0.086
5	Arginine and proline metabolism	37	24	5	1.32	0.32	0.52	0.087
6	Selenocompound metabolism	16	6	2	0.57	0.24	0.64	0.10
7	Drug metabolism - cytochrome P450	43	6	2	1.53	0.24	0.64	0.10
8	Valine, leucine and isoleucine biosynthesis	8	7	2	0.29	0.30	0.69	0.11
9	Tyrosine metabolism	42	37	6	1.50	0.54	0.71	0.11
10	Arginine biosynthesis	14	8	2	0.50	0.36	0.74	0.12
11	Glycine, serine and threonine metabolism	30	17	3	1.07	0.52	0.77	0.12
12	Pyrimidine metabolism	39	10	2	1.39	0.48	0.82	0.13
13	Valine, leucine and isoleucine degradation	35	12	2	1.25	0.59	0.87	0.15
14	Ascorbate and aldarate metabolism	8	5	1	0.29	0.58	1.0	1.0
15	Ubiquinone and other terpenoid-quinone biosynthesis	9	3	1	0.32	0.40	1.0	1.0

Fig. S3. Enrichment analysis output

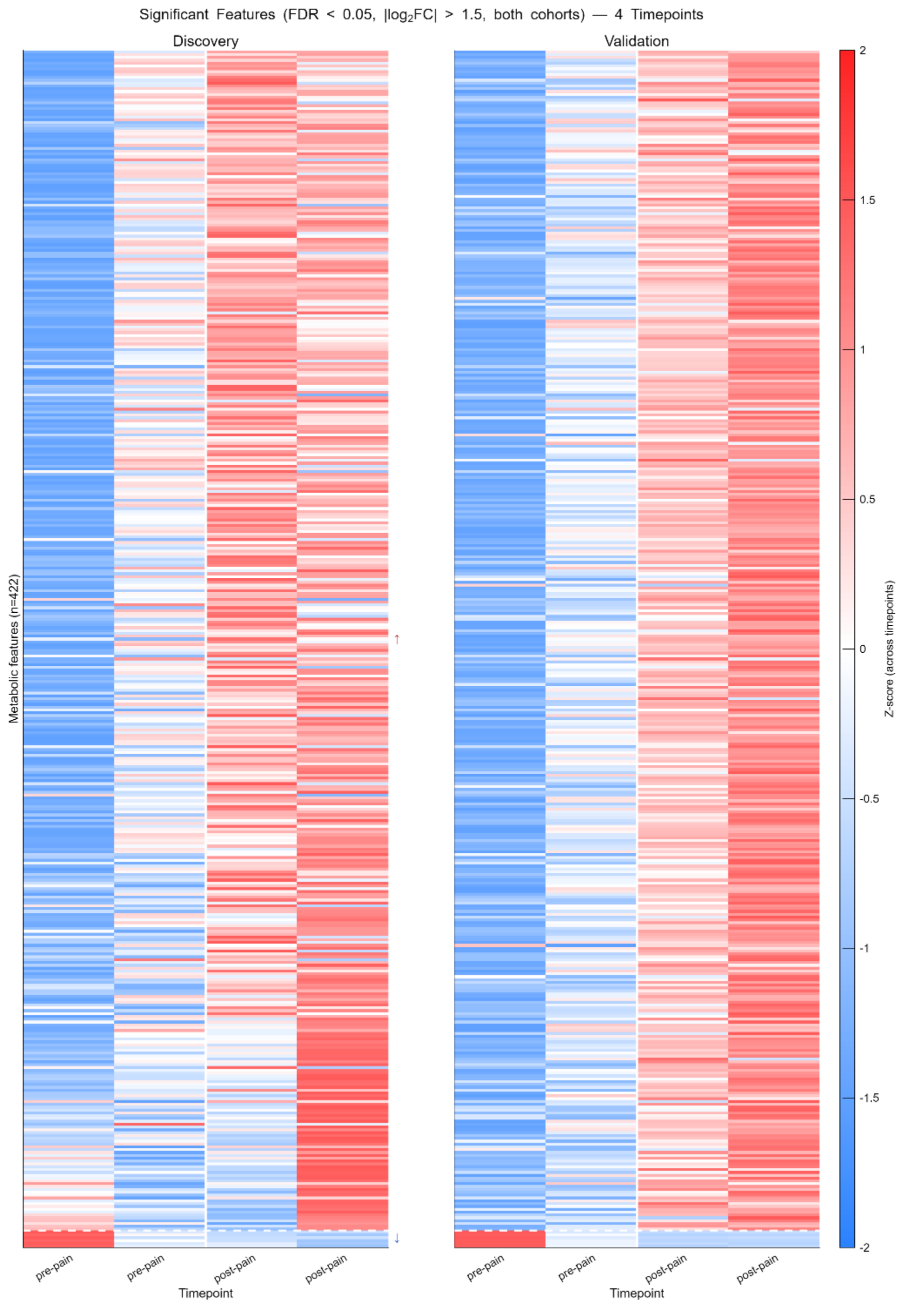

Fig. S4. Heatmap of all 422 significant metabolic features across four measurement timepoints in the Discovery and Validation cohorts.Z-score normalized signal intensities are shown for all metabolic features significant in both cohorts (FDR < 0.05, $|\log_2 FC| > 1.5$) across two pre-CPT baselines (BL1, BL2) and two post-CPT measurements (immediately post, 15 min post). Features are sorted by direction of regulation (upregulated n=416, top; downregulated n=6, bottom) and magnitude of change. Color scale: red = relatively elevated, blue = relatively reduced.
